# Advanced gastric cancer with features of a submucosal tumor diagnosed by endoscopic ultrasound-guided fine needle aspiration and boring biopsy preoperatively: A case report and literature review

**DOI:** 10.1016/j.ijscr.2019.01.044

**Published:** 2019-02-08

**Authors:** Hiroaki Yamane, Michihiro Ishida, Seisyu Banzai, Tetsushi Kubota, Soichiro Miyake, Yasuhiro Choda, Hitoshi Idani, Shigehiro Shiozaki, Masazumi Okajima

**Affiliations:** a Department of Surgery, Hiroshima City Hiroshima Citizens Hospital, Hiroshima, Japan; b Department of Pathology, Hiroshima City Hiroshima Citizens Hospital, Hiroshima, Japan; c Department of Gastroenterological and Transplant Surgery, Graduate School of Biomedical and Health Sciences, Hiroshima University, Hiroshima, Japan

**Keywords:** Endoscopic ultrasound-guided fine needle aspiration, Gastric cancer, Submucosal tumor

## Abstract

**Introduction:**

Gastric cancer with features of a submucosal tumor (GCSMT) is rare, and the preoperative diagnosis is very difficult. We present a case of GCSMT diagnosed by endoscopic ultrasound-guided fine needle aspiration (EUS-FNA) and boring biopsy (deeper biopsy), successfully treated with distal gastrectomy and lymph node dissection.

**Presentation of case:**

An 81-year-old man with a history of myocardial infarction and type 2 diabetes mellitus was admitted to our hospital for further examination of a gastric submucosal tumor. Endoscopic examination of the gastrointestinal tract showed a 30-mm submucosal tumor at the lower gastric body. Enhanced computed tomography revealed a tumor located at the lower body of the stomach and pyloric lymph node swelling. EUS-FNA and boring biopsy from the tumor revealed adenocarcinoma. Accordingly, distal gastrectomy with lymph node dissection was performed. Histopathological examination showed a poorly differentiated adenocarcinoma. Postoperative diagnosis was GCSMT with lymph node metastasis (T2, N1, M0, stage IIA).

**Conclusion:**

We report a rare case of GCSMT with lymph node metastasis. Preoperative diagnosis of GCSMT is necessary to guide surgical management. EUS-FNA and boring biopsy could aid in obtaining adequate quantities of the specimens for histopathological and immunohistochemical diagnosis.

## Highlights

• Gastric carcinoma with features of a submucosal tumor is a rare condition.
• Gastric carcinoma with features of a submucosal tumor has unique features.
• Tissue sampling must be performed if gastric malignant submucosal tumor is suspected.

## 1 Introduction

Gastric cancer is the fourth most common malignancy worldwide [1]. Endoscopic examination and biopsy from the surface of the tumor are essential for the diagnosis because most gastric cancers are derived from the mucosal epithelial cells. However, the preoperative diagnosis of gastric cancer presenting as submucosal tumor (SMT) is difficult, as it is typically covered with non-malignant epithelial mucosa [2]. Moreover, the clinical and pathological characteristics of gastric carcinoma with features of a submucosal tumor (GCSMT) remain unclear because of the limited number of reported cases [2,3]. We report a rare case of GCSMT diagnosed preoperatively by endoscopic ultrasound-guided fine needle aspiration (EUS-FNA) and boring biopsy (deeper biopsy). Boring biopsy is a method of digging into the gastric mucosa using a forceps to obtain the tissue specimens of the gastric SMT. We also conduct a literature review on GCSMT. This case has been reported in line with the SCARE criteria [4].

## 2 Case presentation

**Fig. 1:**
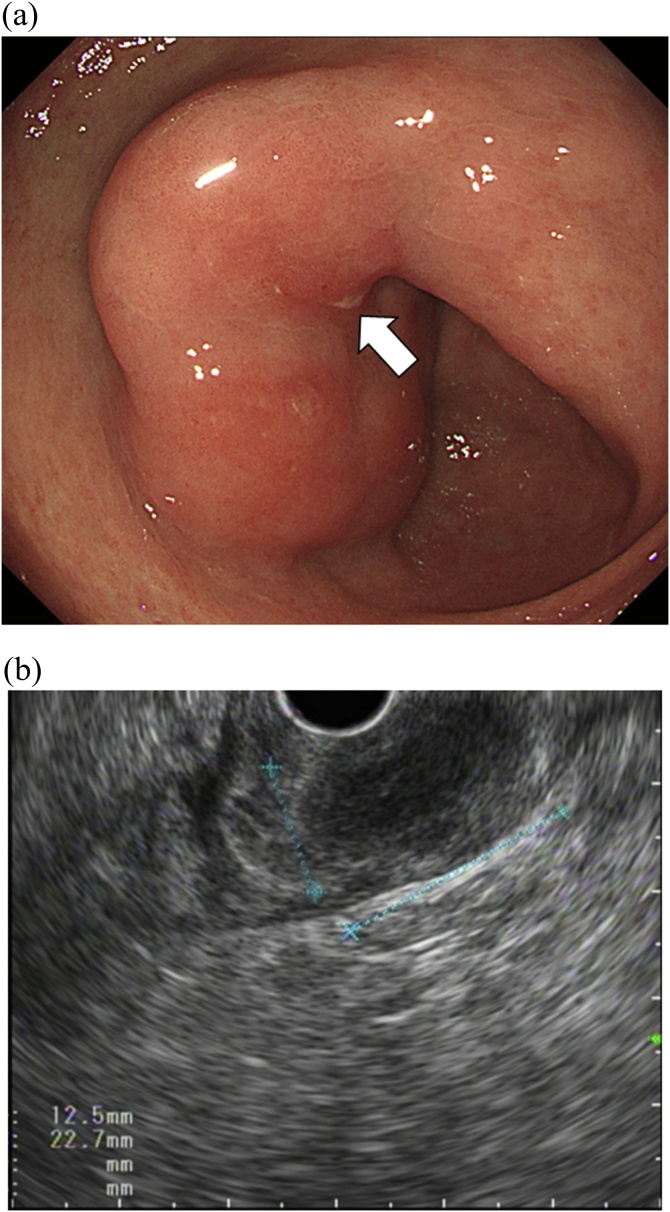
Preoperative endoscopic examination and endoscopic ultrasonography. (A) Endoscopic image of the submucosal tumor. The surface of the lesion is covered by non-malignant mucosa with erosion (arrow). (B) Endoscopic ultrasound examination showed a well-defined hypoechoic mass (22 × 12 mm), which originates from the submucosal layer of the stomach.

An 81-year-old man was admitted to our hospital because of gastric SMT that was noted during an annual gastrointestinal examination. He had a history of type 2 diabetes mellitus and myocardial infraction treated with anticoagulant therapy, percutaneous intervention, and coronary artery bypass graft. His blood examination results were within normal range, but the tumor marker levels were high: carcinoembryonic antigen, 16.8 ng/mL (normal, <5.0 ng/mL) and α-fetoprotein (AFP), 83.5 ng/mL (normal, <10.0 ng/mL). Endoscopic examination of the gastrointestinal tract revealed SMT (approximately 30 mm in diameter) located at the lower gastric body and with erosion on the top of the tumor (Fig. 1A). Endoscopic ultrasonography (EUS) revealed that the tumor presented as a well-defined hypoechoic mass (22 × 12 mm) arising from the submucosal layer of the stomach (Fig. 1B). Endoscopic biopsy of the erosion site revealed no malignancy. EUS-FNA and boring biopsy were performed. Histopathological evaluation revealed that the tumor had dense proliferation of larger atypical cells. Immunohistochemical analysis revealed that these tumor cells were positive for Caudal-type homeobox-2 (CDX-2) and negative for synaptophysin and chromogranin A. These findings suggested that the SMT originated from a gastrointestinal adenocarcinoma. Contrast-enhanced computed tomography revealed a 30-mm tumor located in the gastric vestibule and an enlarged lymph node No. 6 (22 mm). Hence, the preoperative diagnosis was gastric carcinoma with lymph node metastasis.

**Fig. 2:**
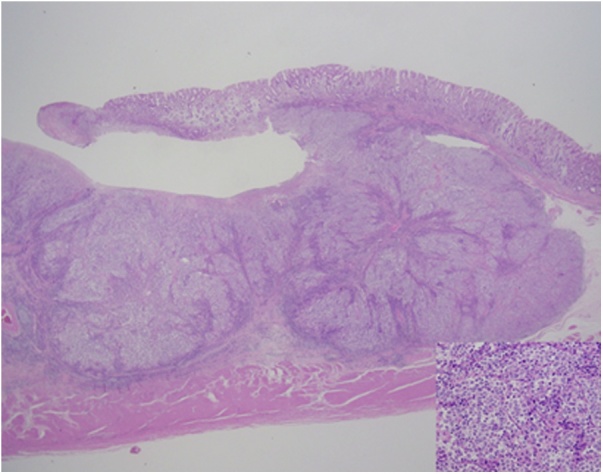
Histological features. Components of a poorly differentiated adenocarcinoma invaded the muscularis propria. The tumor was almost entirely covered with non-malignant epithelial mucosa.

We performed distal gastrectomy with D2 lymph node dissection and Roux-en-Y reconstruction after obtaining informed consent from the patient. The resected specimen showed a tumor (35 × 18 mm) located at the lower body of the stomach. Postoperative histopathological examination of the specimen revealed a poorly differentiated adenocarcinoma with high lymphocyte proliferation in the peripheral tumor. Although the tumor invaded the muscularis propria, the tumor surface was covered with non-malignant epithelial mucosa (Fig. 2). Moreover, immunohistochemical analysis demonstrated that napsin and AFP were negatively expressed and CDX-2 was diffusely expressed. Epstein-Barr virus (EBV)-encoded RNA-1 (EBER-1) in situ hybridization confirmed the absence of EBV in gastric tumor cells. Metastasis of the adenocarcinoma was found in 1 (No. 6) of 41 excised lymph nodes. The postoperative pathological stage was IIA (T2N1cM0), according to the Japanese classification of gastric cancer [5].

The patient was discharged on the postoperative day 19 without any complications. Although he refused adjuvant chemotherapy, no recurrence or metastasis occurred during the follow-up period of 18 months.

## 3 Discussion

Gastric SMTs consist of a variety of neoplastic and non-neoplastic conditions arising from deeper layers of the gastric wall, such as gastrointestinal stromal tumors (GIST), schwannomas and malignant lymphomas [6]. GCSMT is a rare disease with an incidence of 0.1 to 0.63% of all gastric cancer [2]. The clinical and pathological findings of these lesions remain unclear because of a few numbers of reported cases. Herein, we reviewed 19 cases (17 case reports) published in English, including our case, in order to elucidate the clinical characteristics of GCSMT (Table 1) [2,3,[7], [8], [9], [10], [11], [12], [13], [14], [15], [16], [17], [18], [19], [20], [21]].

**Table 1:** Previous case reports of gastric cancer resembling submucosal tumor following curative surgery.

Author	Age	Sex	Tumor location	Maximal tumor size	Endoscopic resection	Surgery	TNM classification^a^	Type of tissue
Ohara	48	Male	L	20 mm		Sub TG	3, 0, 0, II	Well
Hosoda	71	Male	M	12 mm		SubTG	1b, 2, 0, IA	Poorly
Umehara	50	Male	U	35 mm		TG	2, 0, 0, IB	Poorly
Kume	49	Female	M	10 mm	○	Sub TG	1b, 0, 0, IA	Well
Fujiyoshi	73	Female	U	10 mm		PG	1b, 0, 0, IA	Moderately
Takahashi	50	Male	L	20 mm		DG	2, 0, 0, IB	LELC
Teraishi	63	Male	L	25 mm		DG	2, 0, 0, IB	Moderately
Ando	65	Female	M	20 mm		Sub TG	1b, 0, 0, IA	Mucinous
Kim	66	Male	U	73 mm		TG	2, 0, 0, IB	Mucinous
Kim	46	Male	U	25 mm		TG	1b, 0, 0, IA	LELC
Yu	54	Female	L	31 mm		Laparoscopic partial gastrectomy	3, X, 0, IIA	Mucinous
Yu	50	Female	M	50 mm		TG	4a, 2, 0, IIIB	Mucinous
Yoo	40	Female	U	25 mm		TG	1b, 0, 0, IA	Mucinous
Matsumoto	58	Male	U	ND	○	Radial gastrectomy	1b, 0, 0, IA	LELC
Imamura	68	Male	U	20 mm		Laparoscopic PG	1b, 0, 0, IA	Well
Cha	69	Male	U	24 mm		TG	ND	Fundic gland
Chen	50	Male	M	22 mm	○	TG	1b, 0, 0, IA	LELC
Li	44	Male	U	10 mm	○	Radial gastrectomy	1b, 0, 0, IA	Poorly
Present case	81	Male	L	35 mm		DG	2, 1, 0, IIA	Poorly

Abbreviations: L, lower body of the stomach; LELC, lymphoepithelioma-like carcinoma; M, middle body of the stomach; ND, not described; PG, proximal gastrectomy; TG, total gastrectomy; TNM, tumor-node-metastasis; U, upper body of the stomach.

a Japanese Gastric Cancer Association 14th Edition.

Thirteen patients (68.4%) were male and the average age of patients was 57 years (range: 40–81 years). The average tumor size was 26 mm (range: 10–73 mm). The tumor was located in the upper body in 9 patients, middle body in 5 patients, and lower body in 5 patients. Histological diagnoses consisted of poorly differentiated adenocarcinomas in 42.1% (8/19 cases), mucinous adenocarcinomas in 26.3% (5/19 cases), tubular adenocarcinomas in 26.3% (5/19 cases), and a fundic gland carcinoma in 5.3% (1/19 cases). Previous studies have suggested two hypotheses to describe the pathophysiology of GCSMT [22]. One hypothesis is that GCSMT is related with the mass-forming proliferation of cancer cells arising from heterotopic glands under the mucosa. It will be depended on the histological type and the cancer stroma volume, including such as mucinous carcinoma and medullary infiltration type. Another hypothesis is that GCSMT is related with the response of surrounding tissues to cancer invasion, including such as lymphocytic infiltration and local fibrosis. As lymphoepithelioma-like gastric carcinoma, it is characterized by carcinoma with intense stromal lymphocytic infiltration. Epstein-Barr virus (EBV) infection is also closely associated with the lymphocytic infiltration [23].

Our patient had a poorly differentiated adenocarcinoma without lymphocytic cyst and was negative for EBV, consistent with the medullary infiltration type.

In the treatment of GCSMT, preoperative diagnosis is necessary. Because GCSMT is a malignant tumor, its treatment typically requires systemic gastrectomy with en bloc lymphadenectomy [24]. This differs from the treatment of any other SMT [25]. However, a preoperative diagnosis of GCSMT is difficult because the tumor surface is covered with non-malignant epithelium. Therefore, the possibility of malignancy should be considered carefully in all cases of gastric SMT. Lim et al. reported that endoscopic biopsy in patients with gastric SMT appropriately diagnosed malignancy in approximately 33% (16 of 49 cases) [25]. In our reviewed cases, the endoscopic biopsy diagnosis rate of GCSMT was 47.4% of all actual cases (9 of 19 cases). In another 7 cases, despite negative endoscopic biopsy results, gastrectomy was performed owing to suspected malignancy (3 cases underwent intraoperative frozen section examinations; 2 cases underwent gastrectomy with lymphadenectomy; 1 case underwent partial gastrectomy without lymphadenectomy; and 1 case underwent laparoscopy and endoscopy cooperative surgery before curative resection). In the other 4 cases, endoscopic resection was performed to determine a definitive diagnosis, after which an additional radical cure excision was performed. Indeed, following definitive diagnoses, 18 cases underwent a systematic gastrectomy with an en bloc lymphadenectomy as an initial treatment.

EUS is a useful tool for the diagnosis of gastric diseases with an accuracy rate ranging from 45.5 to 66.7% [25,26]. EUS could provide information on the tumor and its surrounding structures, as well as visualize the layer of origin of the gastric SMT. Kawamoto et al. reported the features of malignant SMT on EUS as follows: 1) tumor size >30–50 mm, 2) rapid tumor growth, 3) echo-heterogeneity, and 4) irregular margins [27]. In our case, although EUS showed that the gastric tumor had a well-defined hypoechoic pattern, the tumor was arising from the third layer (submucosal layer) of the gastric wall. This is notable because this location differs from that of any other malignant gastric SMT, and especially of gastric GIST, which has malignant potential and which characteristically arises from the fourth layer (muscularis propria) [28]. Additional features which raise the suspicion of a malignancy GCSMT are elevated tumor markers and enlarged regional lymph nodes.

Finally, histopathological evaluation of the tumor with tissue sampling is essential to confirm the diagnosis. EUS-FNA is a less-invasive method and more effective for gastric SMT, with an accuracy of approximately 80% [25,28]. Moreover, our patient underwent boring biopsy, with which adequate quantities of specimens from the tumor could be obtained for histopathological and immunohistochemical analysis. Adequate tissue sampling by EUS-FNA and boring biopsy could aid in establishing the accurate preoperative diagnosis, which, in turn, could help clinicians in selecting an optimal treatment strategy for the patients.

## 4 Conclusion

We reported a rare case of GCSMT with lymph node metastasis, which was diagnosed preoperatively. Based on our experience and results of the literature review, we recommend that in lesions suspected to be malignant gastric submucosal tumor, tissue sampling should be performed proactively, because the treatment strategy for GCSMT is quite a different from that for any other gastric SMT. For tissue sampling, EUS-FNA and boring biopsy are useful and minimally invasive. Finally, further studies including more cases are warranted to improve our knowledge of this important diagnosis and guide future clinical practice.

## Conflicts of interest

None of the authors have any commercial or financial involvement in connection with this study that represents or appears to represent any conflicts of interest.

## Sources of funding

This research received no specific grant from any funding agency in the public, commercial, or not-for-profit sectors.

## Ethical approval

This research was conducted in accordance with the regulations of the Hiroshima City Hiroshima citizens hospital ethics committee.

## Consent

Written informed consent was obtained from the patient for publication of this case report and any accompanying images.

## Author’s contribution

Hiroaki Yamane was involved in writing the report; Michihiro Ishida designed the study and was involved in writing the report; and Seisyu Banzai, Tetsushi Kubota, Soichiro Miyake, Yasuhiro Choda, Hitoshi Idani, Shigehiro Shiozaki, and Masazumi Okajima revised the report critically for important intellectual content.

## Registration of research studies

Researchregistry4517.

## Guarantor

Michihiro Ishida.

## Provenance and peer review

Not commissioned, externally peer-reviewed.

## References

[1] Torre L.A., Bray F., Siegel R.L., Ferlay J., Lortet-Tieulent J., Jemal A. (2015). Global cancer statistics, 2012. CA Cancer J. Clin..

[2] Umehara Y., Kimura T., Okubo T., Sano Y., Nakai K., Oi S. (1999). Gastric carcinoma resembling submucosal tumor. Gastric Cancer.

[3] Fujiyoshi A., Kawamura M., Ishitsuka S. (2003). Gastric adenocarcinoma mimicking a submucosal tumor: case report. Gastrointest. Endosc..

[4] Agha R.A., Fowler A.J., Saeta A., Barai I., Rajmohan S., Orgill D.P. (2016). The SCARE statement: consensus-based surgical case report guidelines. Int. J. Surg..

[5] Japanese Gastric Cancer Association (2011). Japanese classification of gastric carcinoma: 3rd English edition. Gastric Cancer.

[6] Guo J., Liu Z., Sun S., Wang S., Ge N., Liu X. (2013). Endosonography-assisted diagnosis and therapy of gastrointestinal submucosal tumors. Endosc. Ultrasound.

[7] Ohara N., Tominaga O., Uchiyama M., Nakano H. (1997). A case of advanced gastric cancer resembling submucosal tumor of the stomach. Jpn. J. Clin. Oncol..

[8] Hosoda Y., Hanai H., Arai H., Sato Y., Kajimura M., Sugimura H. (1999). Early gastric cancer exhibiting the features of submucosal tumor: report of a case. Endoscopy.

[9] Kume K., Yoshikawa I., Yamazaki M., Abe S., Murata I., Otsuki M. (2001). A case of gastric cancer with features of submucosal tumor. Gastrointest. Endosc..

[10] Takahashi T., Otani Y., Yoshida M., Furukawa T., Kameyama K., Akiba Y. (2005). Gastric cancer mimicking a submucosal tumor diagnosed by laparoscopic excision biopsy. J. Laparoendosc. Adv. Surg. Tech. A.

[11] Teraishi F., Uno F., Kagawa S., Fujiwara T., Gouchi A., Tanaka N. (2007). Advanced gastric adenocarcinoma mimicking a submucosal tumor. Endoscopy.

[12] Ando H., Morinaga N., Shitara Y., Suzuki K., Toyoda M., Kano H. (2008). Gastric cancer with features of submucosal tumor: a case report. Hepatogastroenterology.

[13] Kim K.Y., Kim G.H., Heo J., Kang D.H., Song G.A., Cho M. (2009). Submucosal tumor-like mucinous gastric adenocarcinoma showing mucin waterfall. Gastrointest. Endosc..

[14] Kim H.G., Ryu S.Y., Yun S.K., Joo J.K., Lee J.H., Kim D.Y. (2012). Preoperative predictors of malignant gastric submucosal tumor. J. Korean Surg. Soc..

[15] Yu B.C., Lee W.K. (2013). Two cases of mucinous adenocarcinoma of the stomach mistaken as submucosal tumor. J. Korean Surg. Soc..

[16] Yoo C.H., Park S.J., Park M.I., Moon W., Kim H.H., Lee J.S. (2013). Submucosal tumor-like early-stage mucinous gastric carcinoma: a case study. Korean J. Gastroenterol..

[17] Matsumoto T., Shimeno N., Imai Y., Inokuma T. (2013). Gastric carcinoma with lymphoid stroma resembling a hypoechoic submucosal tumor. Gastrointest. Endosc..

[18] Imamura T., Komatsu S., Ichikawa D., Kobayashi H., Miyamae M., Hirajima S. (2015). Gastric carcinoma originating from the heterotopic submucosal gastric gland treated by laparoscopy and endoscopy cooperative surgery. World J. Gastrointest. Oncol..

[19] Cha H.J., Kim K., Kim M., Choi H., Kim Y.M., Suh J.H. (2016). Concurrent gastric adenocarcinoma of fundic gland type and carcinoma with lymphoid stroma: a rare case report. Case Rep. Gastroenterol..

[20] Chen M., Yin L., Yao Y., Wang L., Xu G., Zhang X. (2016). Lymphoepithelioma-like gastric carcinoma in a patient with rectal laterally spreading tumor: a case report. Oncol. Lett..

[21] Li L., Lian J., Tseng Y., Chen S. (2016). Early gastric cancer presenting as a submucosal tumor. Clin. Gastroenterol. Hepatol..

[22] Yamaoku K., Kunisaki C., Sato T., Oshima T., Fujii S., Noawa A. (2011). A case of gastric cancer resembling a submucosal tumor difficulty to diagnose preoperative with a review of 183 cases. J. Jpn. Coll. Surg..

[23] Tokunaga M., Land C.E., Uemura Y., Tokudome T., Tanaka S., Sato E. (2017). Epstein-Barr virus in gastric carcinoma. Am. J. Pathol..

[24] Japanese Gastric Cancer Association (2017). Japanese gastric cancer treatment guidelines 2014 (ver. 4). Gastric Cancer.

[25] Lim T.W., Choi C.W., Kang D.H., Kim H.W., Park S.B., Kim S.J. (2016). Endoscopic ultrasound without tissue acquisition has poor accuracy for diagnosing gastric subepithelial tumors. Medicine (Baltimore).

[26] Karaca C., Turner B.G., Cizginer S., Forcione D., Brugge W. (2010). Accuracy of EUS in the evaluation of small gastric subepithelial lesions. Gastrointest. Endosc..

[27] Kawamoto K., Yamada Y., Utsunomiya T., Okamura H., Mizuguchi M., Motooka M. (1997). Gastrointestinal submucosal tumors: evaluation with endoscopic US. Radiology.

[28] Hoda K.M., Rodriguez S.A., Faigel D.O. (2009). EUS-guided sampling of suspected GI stromal tumors. Gastrointest. Endosc..

